# Supplementary material for: The Evolution and Characterization of the RNA Interference Pathways in Lophotrochozoa
Source: Genome Biol Evol. 2024 May 7;16(5):evae098. doi: 10.1093/gbe/evae098 (PMC11114477; doi:10.1093/gbe/evae098)
Supplement: evae098_Supplementary_Data [file evae098_supplementary_data.zip › Supplementary_materials.zip!/selected_miRNAs/NoGe_selected_miRNAs/known_miRNA_families_pdf_files/NMRB01001213.1_Notospermus_geniculatus_strain_Ushimado_scaffold1213,_whole_genome_shotgun_sequence_26251.pdf]

Provisional ID	:	NMRB01001213.1_Notospermus_geniculatus_strain_Ushimado_scaffold1213,_whole_genome_shotgun_sequence_26251
Score total	:	21577.7
Score for star read(s)	:	3.9
Score for read counts	:	21569.3
Score for mfe	:	1.5
Score for randfold	:	0
Score for cons. seed	:	3
Total read count	:	42319
Mature read count	:	42195
Loop read count	:	0
Star read count	:	124

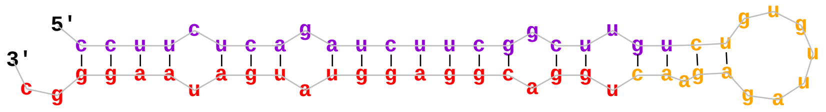

Genome coordinates: NMRB01001213.1 42787-42845

pre-miRNA sequence:
CCUUCUCAGAUCUUCGGCUUGUCCUUCUCAGAUCUUCGGCUUGUCUGUGUUAGAGAACUGGACGGAGGUAUGAUAAGGGC

Mature sequence: UGGACGGAGGUAUGAUAAGGGC

Aligned mature sequence of the *N. geniculatus* pre-miRNA with the top 5 hits from the BLAST search against the pre-miRNA mirGeneDB set:

```
                             *********   **********
Nge_Mir-184-P6               uggacggagguaugauaagggc
Lgi-Mir-184-P6_3p            uggacggagaacugauaagggc
```
